# Otopathogenic *Staphylococcus aureus* Invades Human Middle Ear Epithelial Cells Primarily through Cholesterol Dependent Pathway

**DOI:** 10.1038/s41598-019-47079-7

**Published:** 2019-07-25

**Authors:** Rahul Mittal, Luca H. Debs, Amit P. Patel, Desiree Nguyen, Patricia Blackwelder, Denise Yan, Paulo H. Weckwerth, Xue Zhong Liu

**Affiliations:** 1 Department of Otolaryngology, University of Miami-Miller School of Medicine, Miami, FL USA; 2 Center for Advanced Microscopy, Chemistry Department, University of Miami, Coral Gables, FL USA; 3 RSMAS, University of Miami, Key Biscayne, FL USA; 4 Health Sciences Department, University of Sagrado Coração, Bauru, SP Brazil

**Keywords:** Bacteriology, Pathogens

## Abstract

Chronic suppurative otitis media (CSOM) is one of the most common infectious diseases of the middle ear especially affecting children, leading to delay in language development and communication. Although *Staphylococcus aureus* is the most common pathogen associated with CSOM, its interaction with middle ear epithelial cells is not well known. In the present study, we observed that otopathogenic *S. aureus* has the ability to invade human middle ear epithelial cells (HMEECs) in a dose and time dependent manner. Scanning electron microscopy demonstrated time dependent increase in the number of *S. aureus* on the surface of HMEECs. We observed that otopathogenic *S. aureus* primarily employs a cholesterol dependent pathway to colonize HMEECs. In agreement with these findings, confocal microscopy showed that *S. aureus* colocalized with lipid rafts in HMEECs. The results of the present study provide new insights into the pathogenesis of *S. aureus* induced CSOM. The availability of *in vitro* cell culture model will pave the way to develop novel effective treatment modalities for CSOM beyond antibiotic therapy.

## Introduction

Otitis media (OM) refers to infections of the middle ear and can be broadly classified into acute and chronic OM^1–3^. While the molecular mechanisms leading to acute OM are well characterized, the pathophysiology of chronic otitis media (COM) is not fully understood. COM is diagnosed among 31 million new patients each year^4^. One of the most common forms of COM is manifested as chronic suppurative otitis media (CSOM)^5^. In CSOM, there is a perforation of tympanic membrane, presence of ear discharge and recurrent ear infections. The inflammatory mediators produced during CSOM can damage sensory cells in the inner ear leading to hearing loss^6–13^. In addition, chronic infection of the middle ear can lead to edema of the middle-ear lining and possibly ossicular chain disruption that further aggravates the problem of hearing loss in CSOM patients^14^. CSOM remains a serious public health concern in both developing regions such as in Africa, Asia, and Latin America, as well as in developed countries^15^. The World Health Organization (WHO) reports that about 65 to 330 million people worldwide develop CSOM and approximately, 60% will have the hearing dysfunction^16^. When seen in children, this could lead to detrimental effects on language, cognitive and psychosocial development. Therefore, CSOM has a significant economic and medical burden on the society^17,18^. Although the exact incidence of CSOM in the United States has not been reported, but it is presumed that 70% of US children are reported to suffer from at least one acute ear infection before 3 years old and in some cases, it progresses to CSOM^17^. During CSOM, the spread of suppuration from ear to proximal structures including brain can cause life-threatening extracranial and intracranial complications such as mastoiditis, meningitis, and sinusitis^19–21^. CSOM patients may also experience persistent otorrhea, otalgia, and pressure sensation leading to serious deterioration in the quality of life of affected individuals.

The binding and subsequent invasion into the host cells by pathogens is one of the prerequisites to induce infection^22–24^. This helps in establishing a niche for colonization beyond the luminal surface into the intracellular compartment that shelters the pathogen from host defenses. This invasion also helps the pathogen to initiate down-stream signaling in host cells^25^. Although there is no consensus, it has been hypothesized that the invasion of middle ear epithelial cells (MEECs) may play a crucial role in the pathogenesis of CSOM. However, the molecular mechanisms that can lead to invasion of MEECs by otopathogens are still not clear.

*Staphylococcus aureus* is the most common gram-positive pathogen associated with CSOM^26,27^. There has been increase in prevalence of *S. aureus* induced CSOM^28^. *S. aureus* is a potent catalase producing bacteria implicated in a wide variety of infections^29–32^. *S. aureus* utilizes lipases, superantigens, exfoliative as well as membrane-acting toxins to induce infections^33^. It has been observed that during interaction with immune cells, *S. aureus* is recognized as an extracellular pathogen and utilizes aggressive mechanisms to avoid phagocytosis and prevent mounting of potent antimicrobial immune responses^34–37^. However, *S. aureus* also act as an intracellular pathogen especially invading non-immune cells that helps in establishing a niche of infection and exerting pathogenic effects^38–42^.

The emergence of antibiotic resistant strains of *S. aureus* and potential ototoxicity of antibiotics has created an immediate incentive to focus research studies in the area of CSOM in order to identify novel therapeutic agents. An incomplete understanding about the pathogenesis of the disease has hindered the development of effective treatment strategies against CSOM. In the present study, we examined the ability of otopathogenic *S. aureus* to invade human middle ear epithelial cells (HMEECs), *in vitro*. We observed that otopathogenic *S. aureus* can invade HMEECs in a time and dose dependent manner that is primarily dependent on cholesterol pathway.

## Results

### Otopathogenic *S. aureus* invades HMEECs

**Figure 1:**
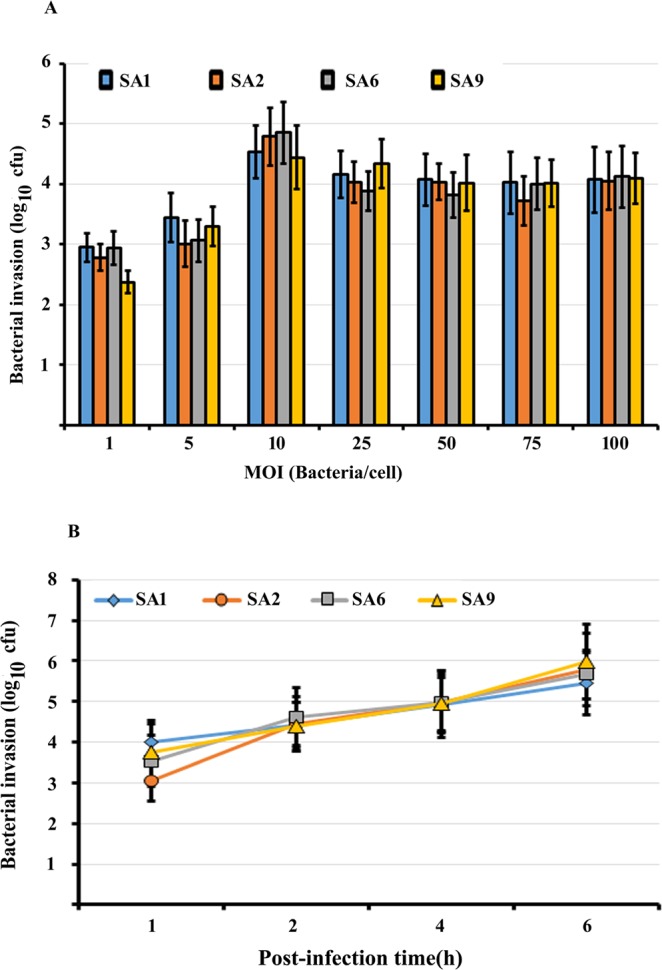
Internalization of otopathogenic *S. aureus* into HMEECs. HMEECs were infected with four clinical strains of *S. aureus* at different multiplicity of infection (MOI) and then subjected to gentamicin and lysostaphin protection assay to determine bacterial cell invasion (**A**). In separate experiments, HMEECs were infected with *S. aureus* at a MOI of 10 for different post-infection time periods and bacterial colonization was determined (**B**). Data represents mean ± standard deviation and is representative of five different experiments carried in triplicate.

To determine whether otopathogenic *S. aureus* can invade HMEECs, we performed the gentamicin and lysostaphin protection assay. Our results indicate that otopathogenic *S. aureus* demonstrates dose and time dependent invasion of HMEECs. Cells were infected with four clinical strains of *S. aureus*, SA1, SA2, SA6 and SA9, at the varying multiplicity of infection (MOI) for 2 h and then subjected to gentamicin protection assay for determining bacterial invasion. At a MOI of 1, log 2.95 colony forming units (cfu) of *S. aureus* strain SA1 were recovered when HMEECs were infected with SA1 for 2 hours. At MOIs of 5, and 10, the cfu increased to log 3.45 and log 4.53 respectively. Further increase in MOI lead to slight decrease in bacterial numbers recovered from HMEECs that can be attributed to steric hinderance. Similar patterns of HMEECs invasion were observed with other otopathogenic *S. aureus* strains, SA2, SA6, and SA9 (Fig. 1A).

Our time dependent study determined the invasion of *S. aureus* at 1 h, 2 h, 4 h, and 6 h post-infection time-periods at a multiplicity of infection (MOI) of 10. While the exact numbers varied from strain to strain, all four strains demonstrated an increase in bacterial numbers inside HMEECs with increase in time-period from 1 h to 6 h. Log 3.99 cfu bacteria were recoverable from HMEECs infected with SA1 for 1 hour. On the other hand, log 5.34 cfu bacteria were demonstrable inside HMEECs by 6 h post-infection. Similar patterns of HMEECs colonization was observed with SA2, SA6 and SA9 strains of otopathogenic *S. aureus* (Fig. 1B). In summary, these results demonstrated *S. aureus* invasion of HMEECs with a logarithmic increase in bacterial numbers across the time points. We were not able to culture any extracellular bacteria following infection of HMEECs and treatment with gentamicin/lysostaphin suggesting that these clinical isolates were completely killed by lysostaphin and gentamicin under the present experimental conditions. To further confirm that intracellular bacteria are susceptible to killing, we treated infected HMEECs (MOI 10, incubation time 2 h) first with gentamicin and lysostaphin to kill extracellular *S. aureus* followed by treatment with cell penetrating antibiotic, minocycline. We observed that minocycline was able to kill intracellular bacteria as we were not able to culture viable *S. aureus* following minocycline treatment (Supplementary Fig. 1). On the other hand, we were able to culture viable *S. aureus* from HMEECs that were not treated with minocycline.

**Figure 2:**
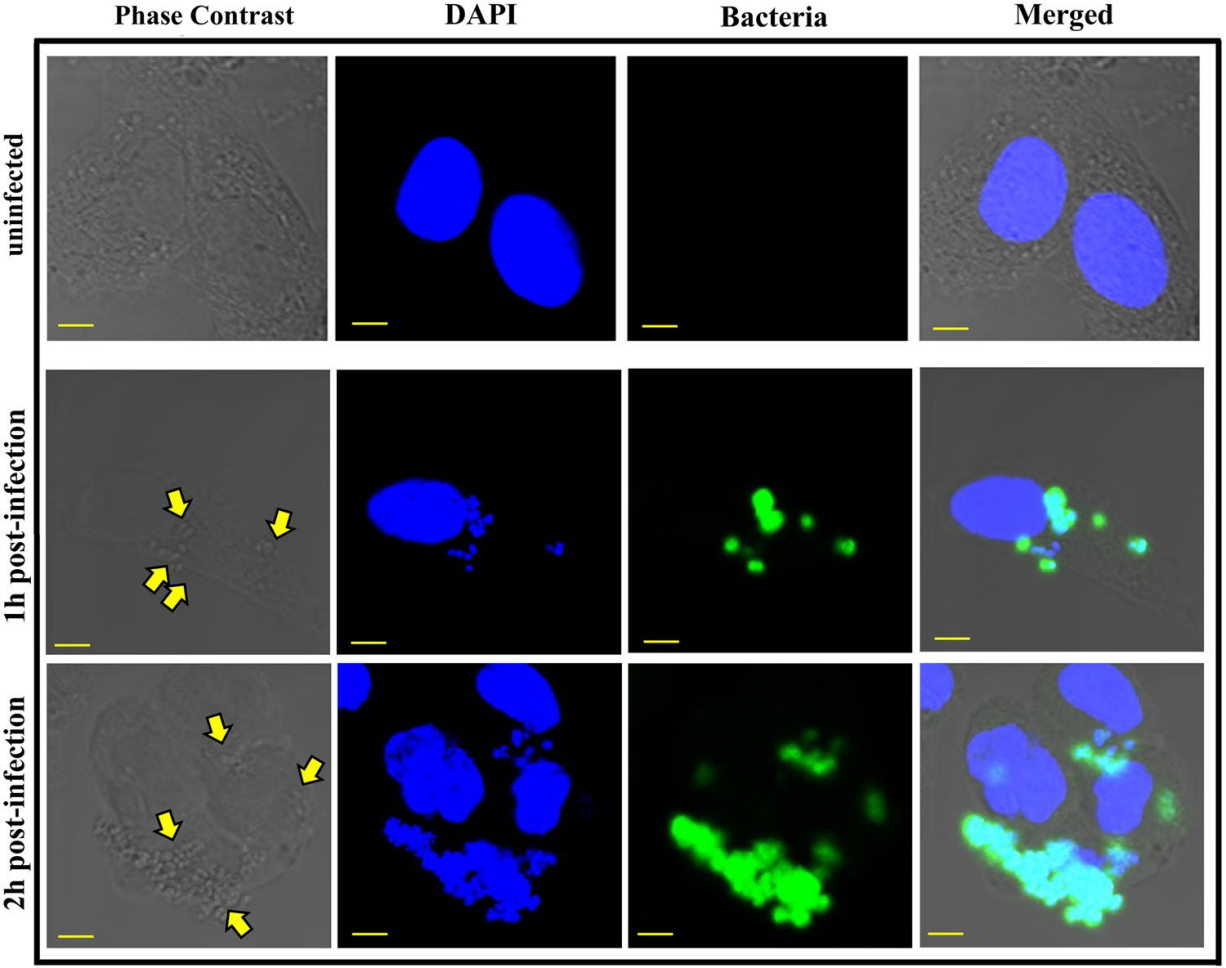
Invasion of HMEECs by *S. aureus* visualized through confocal laser scanning microscopy. HMEECs were left uninfected or infected with *S. aureus* for 1 h and 2 h followed by staining with anti-*S. aureus* antibody. Samples were then stained with a secondary Alexa Fluor^®^ 488 antibody (green) and counterstained with DAPI (blue) to visualize cell nuclei. Cells were then subjected to microscopy. Yellow arrows indicate bacteria. Results are representative of five independent experiments carried out in triplicate. Scale bars: 5 µm.

To confirm the results of our gentamicin protection assay, we subjected *S. aureus* infected HMEECs to confocal scanning laser microscopy. At 1 h post-infection, few bacteria were observed close to the nuclei of the cells confirming cell invasion (Fig. 2). At 2 h post-infection, a large number of bacteria were seen to colonize HMEECs. These results demonstrate that otopathogenic *S. aureus* has the ability to successfully invade HMEECs.

### Scanning electron microscopy of otopathogenic *S. aureus* infected HMEECs

**Figure 3:**
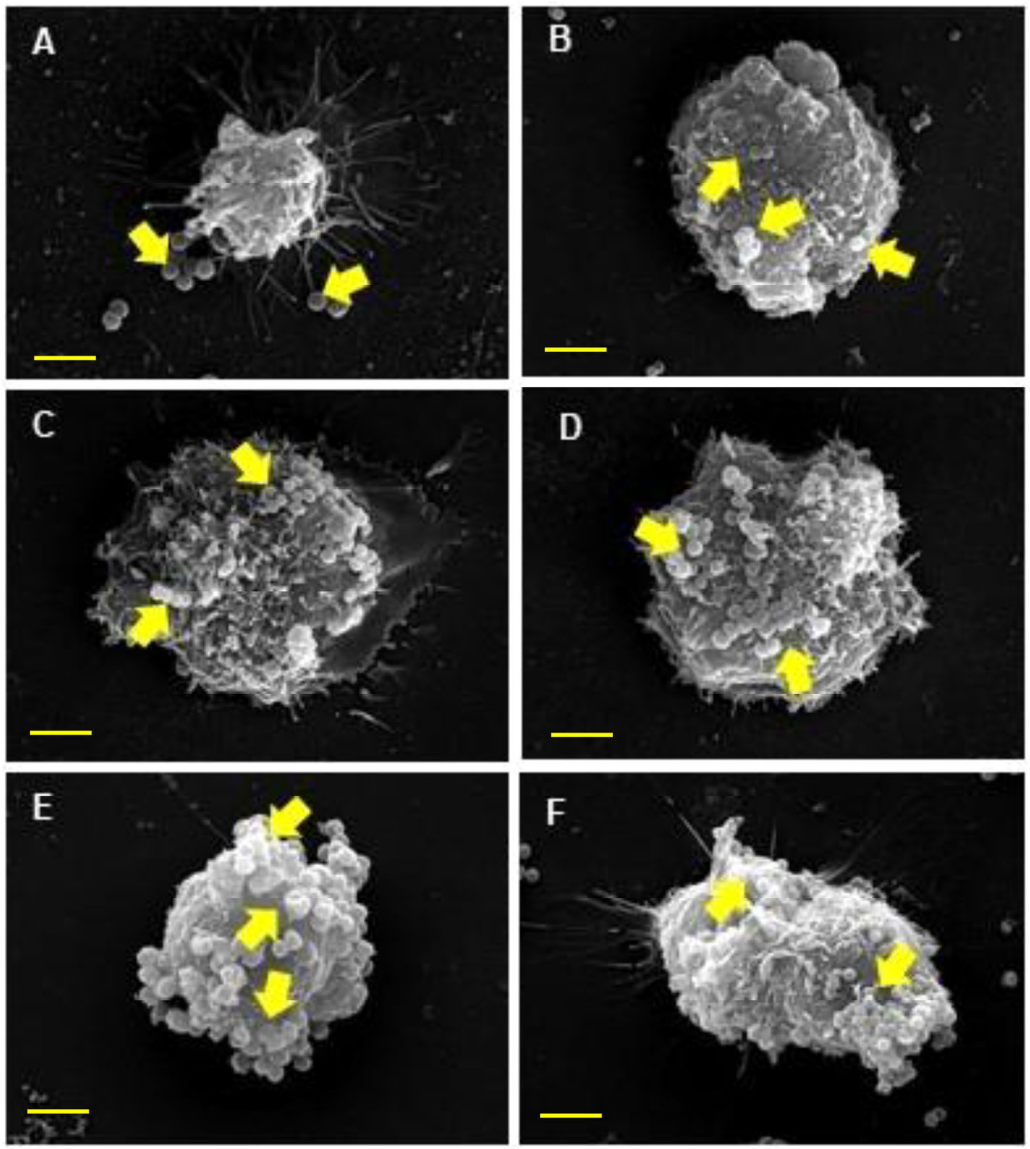
Scanning electron microscopy of HMEECs infected with *S. aureus*. HMEECs were infected with *S. aureus* for 30 min (**A**), 1 h (**B**), 2 h (**C**), 4 h (**D**), 6 h (**E**) and 8 h (**F**). There was increase in number of bacteria on the surface of HMEECs with increase in post-infection time-periods. Yellow arrows indicate bacteria. Scale bars: 5 µm.

The interaction of otopathogenic *S. aureus* with HMEECs was examined in detail using scanning electron microscopy (SEM). By 30 min, we observed loosely attached bacteria on the surface of HMEECs (Fig. 3A). By 1 h post-infection, there few bacteria observable on the surface of HMEECs that further increased in number by 2, 4 and 6 h post-infection (Fig. 3B–E). A large number of bacteria were demonstrable on the surface of HMEECs by 8 h post-infection (Fig. 3F).

### Host pathways involved in otopathogenic *S. aureus* invasion of HMEECs

**Figure 4:**
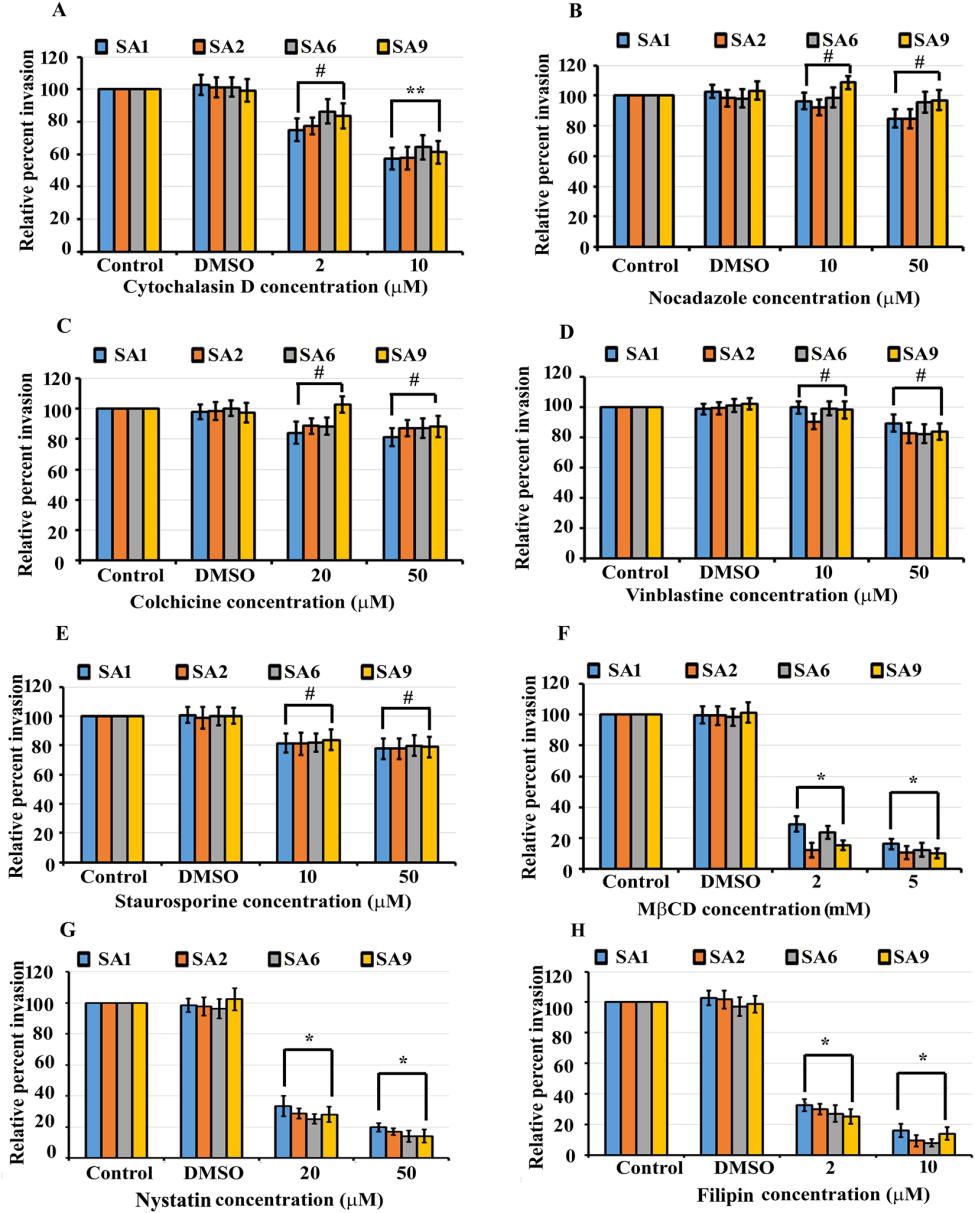
Effect of inhibiting host pathways on invasion of HMEECs by *S. aureus*. HMEECs were pretreated with different doses of cytochlasin D (**A**), nocadazole (**B**), colchicine (**C**), vinblastine (**D**), staurosporine (**E**), MβCD (**F**), nystatin (**G**) and filipin (**H**) followed by infection with *S. aureus*. A significant decrease in invasion was observed when HMEECs were pretreated with MβCD, nystatin, filipin and cytochlasin D. Results are expressed as the percentage of the control group without any inhibitor and represents mean ± standard deviation. Data is representative of four experiments carried out in triplicate. ^#^P > 0.05 or **P < 0.05 or  *P < 0.001 compared to control.

Since host pathways have been implicated in cell invasion by pathogens, we set forth to determine the signaling pathway involved in *S. aureus* invasion of HMEECs. To dissect the host biochemical pathways involved in *S. aureus* cell invasion, HMEECs were pretreated with actin polymerization inhibitor, cytochalasin D, or microtubule disrupting agent, nocodazole, colchicine and vinblastine, protein kinase inhibitor, staurosporine as well as three inhibitors of cholesterol metabolism, methyl-β-cyclodextrin (MβCD), nystatin and filipin, followed by infection with bacteria. Different concentrations of these inhibitors were selected based on previous studies. These inhibitors were dissolved in dimethylsulfoxide (DMSO), therefore, HMEECs treated with DMSO alone served as vehicle control. With increase in concentration of cytochalasin D, there was a significant decrease in the bacterial invasion compared to control cells (Fig. 4A). HMEECs pretreated with 2 µM of cytochalasin D showed an invasion of 75% by *S. aureus* strain SA1 whereas cells pretreated with 10 µM showed invasion of 57.3% compared to control cells (P < 0.05). Inhibition of invasion was less significant when using nocodazole colchicine and vinblastine, each of which induces microtubule disruption. Pretreatment of HMEECs with 10 µM and 50 µM of nocodazole resulted in 96.5% and 84.9% invasion of *S. aureus* strain SA1 relative to control group (P > 0.05), respectively (Fig. 4B). The colchicine and vinblastine pretreated cells showed similar results as those observed with nocodazole. Pretreatment of HMEECs with 20 µM and 50 µM of colchicine resulted in 84.2% and 81.3% invasion relative to control (P > 0.05), respectively (Fig. 4C). Vinblastine pretreated cells showed an invasion of 100% and 89.3% by *S. aureus* at doses of 10 µM and 50 µM, respectively (P > 0.005) (Fig. 4D). Staurosporine was also not able to significantly prevent the internalization of *S. aureus* within HMEECs showing invasion of 81.5% and 77.7% at concentrations of 10 µM and 50 µM, respectively (P > 0.005) (Fig. 4E). Intriguingly, treatment with cholesterol depletion agent, MβCD, at concentrations of 2 mM and 5 mM, reduced invasion of *S. aureus* strain SA1 to 29.1% and 16.1% relative to control cells (P < 0.001), respectively (Fig. 4F). Nystatin and filipin that disrupt lipid raft function were also able to significantly reduce the invasion of HMEECs by *S. aureus* strain SA1 to 19.7% and 15.9% at 50 µM and 10 µM respectively relative to control cells (P < 0.001) (Fig. 4G,H). Similar pattern of HMEECs invasion was observed with three additional strains of *S. aureus*. We observed that there were no toxic effects of these reagents on cells or on bacteria or at the tested concentrations (Supplementary Figs 2 and 3).

### Interaction of otopathogenic *S. aureus* with lipid rafts in HMEECs

**Figure 5:**
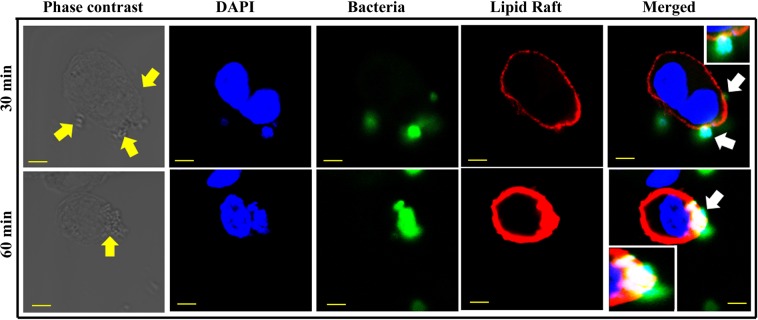
*S. aureus* colocalizes with lipid rafts. HMEECs were infected with *S. aureus* for 30 min and 60 min followed by staining with anti-*S. aureus* antibody and anti-Flotillin 1 antibody to visualize bacteria (green) and lipid rafts (red) respectively. Yellow arrows indicate bacteria and white arrow represent colocalization. Inset shows magnified view. Scale bars: 5 µm.

To confirm our inhibitor treatment data, which suggests that cholesterol plays a crucial role in invasion of HMEECs by *S. aureus*, we stained the infected cells with lipid raft marker and subjected the samples to confocal microscopy. We observed that *S. aureus* colocalizes with lipid rafts in HMEECs. At 30 min post-infection, a few bacteria were found to colocalize with lipid raft at the entry foci (Fig. 5A). At 60 min post-infection, there was an intense lipid raft staining that strongly colocalizes with *S. aureus* (Fig. 5B). These data suggest that otopathogenic *S. aureus* utilizes lipid rafts to invade HMEECs.

## Discussion

CSOM is a recurring infection of the middle ear with associated perforation of the tympanic membrane and subsequent hearing loss^5^. With 31 million new cases of CSOM diagnosed every year, it is imperative that we develop a more robust understanding of the pathophysiology underlying this disease. Research thus far has revealed important features common across cases of CSOM such as invasion of MEECs by otopathogens. However, development of effective treatment modalities beyond antibiotic therapy warrants a thorough understanding of how each otopathogenic bacteria invades MEECs.

*S. aureus* is the most common gram-positive pathogen isolated from CSOM patients^26,27^. However, little is known about the mechanisms or factors involved in invasion of HMEECs by *S. aureus*. In the present study, we showed the colonization of HMEECs by otopathogenic *S. aureus*. The results of gentamicin and lysostaphin protection assay as well as confocal microscopy demonstrated internalization of *S. aureus* within HMEECs. SEM also demonstrated the presence of bacteria on the surface of HMEECs. Our results are in agreement with previous studies which have shown that *S. aureus* is capable of cellular invasion in order to evade environmental stress and host immune responses^35^.

In our preliminary kinetic experiments, the entry of *S. aureus* in HMEECs started at 1 h post-infection with a logarithmic increase in bacterial invasion that positively correlated with post-infection time-period. There was increase in bacterial numbers inside HMEECs with increase in post-infection time-period from 1 h to 6 h. Although limited data is available in published literature regarding kinetics of *S. aureus* entry into host cells, it can persist in host cells up to 7 days^43^. It was observed that all the tested primary and cell lines namely primary human umbilical vein endothelial cells) and EA.hy923 (endothelial cell line), epithelial cells (A549 (lung epithelial cell line) and HaCat (human keratinocyte cell line), osteoblasts (primary human osteoblasts and CRL-11372 (osteoblast cell line)) and connective tissue cells CCD-32-SK (fibroblast cell line) were able to degrade ingested bacteria. However, in all cell types, few bacteria were capable of escaping degradation and persisted intracellularly for up to 7 days^43^. These results are in agreement with our findings that *S. aureus* can survive and persist in host cells.

Bacterial pathogens manipulate host cell pathways for adhesion and invasion into host cells^25^. To dissect the host biochemical pathways involved in cellular invasion by *S. aureus*, HMEECs were treated with inhibitors that block different signaling cascades. The inhibitors utilized were cytochalasin D, staurosporine, nocadazole, colchicine, vinblastine, and MβCD. Cytochalasin D inhibits actin polymerization, while vinblastine nocadazole and colchicine inhibits microtubule formation. Cytochalasin D was able to prevent the internalization of *S. aureus* in HMEECs by about 40% at a dose of 10 µM suggesting that actin polymerization plays some role in cell invasion. However, vinblastine, nocadazole and colchicine has no significant effect in preventing invasion of HMEECs by *S. aureus* suggesting that microtubules plays a little role in cell invasion. The protein kinase inhibitor, staurosporine, was also not able to induce any significant reduction in *S. aureus* colonization of HMEECs. Our results are in agreement with the previous studies. It has been shown that invasion of *S. aureus* into bovine mammary epithelial cells is dependent on cytoskeleton rearrangements, but not on microtubule formation^44^. *S. aureus* also utilizes actin cytoskeleton to invade the human embryonic kidney cell line 293T^45^. Other studies that have also highlighted the crucial role of actin in the host cell invasion by *S. aureus*^46^.

Besides cytoskeletal rearrangements and microtubules, some pathogens have been demonstrated to utilize cholesterol to invade host cells^47,48^. Cholesterol is an important structural component of the cell membrane of vertebrates and is involved in membrane integrity and fluidity^49^. MβCD depletes cholesterol, and thus eliminates the ability of bacteria to invade host cells through this pathway. On the other hand, nystatin and filipin disrupt lipid raft function by precipitating cholesterol in the plasma membrane of the cell^50,51^. In our study, pretreatment of HMEECs with MβCD or nystatin or filipin led to significant inhibition of bacterial cell invasion suggesting that *S. aureus* primarily employs cholesterol pathway to invade HMEECs. The results of previous studies have also suggested that host fatty acids can play a crucial role in the pathogenesis of *S. aureus* induced infections. *S. aureus* can utilize the fatty acids present in host low-density lipoproteins (LDL) to bypass both chemical and genetic inhibition of bacterial fatty acid synthesis^52^. Other pathogens have also been shown to use host cholesterol for cellular invasion. Host cholesterol has been implicated in the uptake of mycobacteria by macrophages^53^. *Helicobacter pylori* also utilizes host cell-derived cholesterol to generate cholesteryl glucosides that are integrated into the bacterial membrane^54,55^. These cholesteryl glucosides contribute to the ability of *H. pylori* to evade phagocytosis, the activation of a T-cell response and thus bacterial clearance^56^. Host cholesterol is also required during the initial phase of colonization by *H. pylori*^57^, as well as in bacterial resistance to antibiotics, antimicrobial peptides and bile salts^58,59^. A number of other pathogens such as *Chlamydia pneumoniae*, *Brucella* spp. and *Francisella tularensis* also utilizes host cholesterol for cell invasion and induce infections^59–65^. The results of the present study and other published literature suggest the crucial role of host cholesterol in bacterial virulence and cell invasion.

Lipid rafts are regions of the plasma membrane with high concentrations of cholesterol and glycolipids bound to the glycosylphosphatidylinositol (GPI) anchored proteins^66^. Generally, these pits are used for endocytosis of important molecules by a variety of cells, but bacteria have evolved the mechanisms to manipulate this pathway to invade cells^67,68^. Bacterial host cell internalization through lipid rafts results in reduced oxidative stress due to the lack of lysosomal fusion involved with transport of lipid rafts. *Pseudomonas aeruginosa*, *Escherichia coli*, and *Shigella flexneri* are some of the bacteria that take advantage of lipid rafts to colonize host cells^69–72^. Lipid rafts can be exploited through a variety of mechanisms, and every bacteria uses a unique approach. *E. coli* utilize an adhesion called FimH at the end of their fimbriae to bind to GPI anchored proteins in lipid rafts^73^. Upon binding, a cascade results in cytoskeletal changes that allow for endocytosis*. P. aeruginosa*, on the other hand, utilizes mechanisms to coalesce lipid rafts into larger rafts to facilitate endocytosis^74^. Besides *P. aeruginosa*, other pathogens such as *Shigella* takes advantage of lipid rafts for host cell colonization. A molecular complex composed of host protein, CD44 the hyaluronan receptor, and *Shigella*, the invasin IpaB, has been demonstrated within lipid rafts^75^. In the present study, we observed colocalization of *S. aureus* within lipid rafts in HMEECs. These results along with reduced bacterial cell invasion following cholesterol depletion suggests that otopathogenic *S. aureus* utilizes lipid rafts to colonize HMEECs. Further studies are warranted to decipher the molecular mechanisms underlying HMEECs invasion by otopathogenic *S. aureus* using lipid rafts.

In summary, our results demonstrate for the first time that *S. aureus* is able to invade HMEECs that can contribute to its capability to induce CSOM by evading host immune responses. Our data also suggest that cholesterol and lipid rafts play an important role in internalization of *S. aureus* by HMEECs. Therefore, decreasing host cholesterol levels by dietary or pharmacological means may have implications in the treatment of CSOM and other bacterial infections. The findings of the present study significantly increase our understanding about the pathogenesis of *S. aureus* induced CSOM. Further investigations employing experimental animal models will help in confirming our *in vitro* findings. In addition, further studies with larger number of isolates are warranted to determine whether the bacterial strains used in the present study are etiological representatives of CSOM causative *S. aureus*. The availability of *in vitro* cell culture models will help in screening novel drugs for CSOM and will open up avenues for developing effective therapeutic modalities for CSOM.

## Materials and Methods

### Cell culture

HMEECs (kindly provided by Dr. David Lim) were generated from human middle ear mucosa as described earlier^76^. HMEECs were cultured and maintained as described earlier^76,77^. Briefly, HMEECs were cultured in a 1:1 mixture of Bronchial Epithelial Cell Basal Medium (Lonza, Allendale, NJ) and Dulbecco’s Modified Eagle Medium (Cellgro, Manassas, VA) supplemented with bronchial epithelial growth medium (BEGM) Singlequots (Lonza, Allendale, NJ) and 10% fetal bovine serum (Life Technologies, Carlsbad, CA). In separate experiments, HMEECs were pretreated with different concentrations of actin polymerization (cytochalasin D) or microtubule disrupting agents (nocodazole, colchicine and vinblastine), a protein kinase inhibitor (staurosporine) or inhibitors of cholesterol metabolism (methyl-β-cyclodextrin, nystatin, filipin) (all inhibitors from Sigma, St. Louis, MO) for 1 h and then subjected to invasion assay.

### Effect of inhibitors on cell viability and bacteria

To determine the effect of inhibitors used in this study on cell viability, HMEECs were incubated with different concentrations of inhibitors for 2 h and then stained with LIVE/DEAD cell viability kit (Thermofisher Scientific, Waltham, MA) as per manufacturer’s instructions. After staining, samples were examined using Zeiss LSM-710 laser scanning microscope. Live cells stained green whereas dead cells appeared red under the confocal microscope. The number of live and dead cells were calculated and results were expressed as percentage cell viability.

The effect of inhibitors on bacterial viability was determined by incubating *S. aureus* with different concentrations of inhibitors and then stained with LIVE/DEAD *BacLight* bacterial viability kit as per manufacturer’s instructions (Thermofisher Scientific, Waltham, MA). Samples were examined using Zeiss LSM-710 laser scanning microscope. Live bacteria stained green whereas dead bacteria appeared red under the confocal microscope. The number of live and dead bacteria were calculated and results were expressed as percentage cell viability.

### Bacterial strains

The four clinical strains of *S. aureus* (SA1, SA2, SA6 and SA9) isolated from CSOM patients were used in this study as described in a previous study^78^. The isolation and identification of *S. aureus* was performed using standard methods^79,80^. Bacteria were grown overnight at 37 °C in a tryptic soy broth (TSB) (Teknova, Hollister, CA) in a rotary shaker.

### Scanning electron microscopy

HMEECs were cultured on glass cover slips and were infected with bacteria for varying time periods. After incubation, the cells were washed 5 times with warm phosphate buffered saline (PBS, pH 7.4, Cellgro, Manassas, VA)) buffer to remove unbound bacteria and were then processed for SEM. Samples were fixed in 2% glutaraldehyde (Electron Microscopy Sciences, Hatfield, PA) in PBS buffer followed by three changes of PBS buffer for 10 min each. The samples were then post–fixed in 1% osmium tetroxide (Electron Microscopy Sciences, Hatfield, PA) in PBS buffer for 45 min and rinsed in three changes of PBS buffer for 10 min each. The samples were dehydrated in a graded series of ethanol, dried in hexamethyldisilazane (HMDS) (Electron Microscopy Sciences, Hatfield, PA) and mounted on carbon adhesive tabs fixed to metal stubs. The samples were coated with palladium in a plasma sputter coater and viewed in a scanning electron microscope (FEI, ESEM-FEG XL-30).

### Invasion assay

To determine colonization of HMEECs by otopathogenic *S. aureus*, we performed gentamicin and lysostaphin protection assay^81^. HMEECs were infected with otopathogenic *S. aureus* at various MOI and for different time periods. Following incubation, cells were washed five times with warm RPMI-1640 medium. After washing, medium containing gentamicin (200 µg/ml) (Sigma, St. Louis, MO) and lysostaphin (5 µg/ml) (Sigma, St. Louis, MO) was added to kill extracellular bacteria and incubated at 37 °C (5% CO_2_) for 1 h. HMEECs were then lysed with 1% saponin (Sigma, St. Louis, MO) to release intracellular bacteria, serially diluted and plated onto tryptic soy agar (TSA) plates (Teknova, Hollister, CA). Bacterial colonies were counted next day after incubation overnight at 37 °C.

In some experiments, HMEECs were infected with *S. aureus* at a MOI of 10 for 2 h followed by killing of extracellular bacteria using gentamicin and lysostaphin. Cells were then incubated with cell penetrating antibiotic, minocycline, for 1 h or left untreated. After incubation, cells were washed followed by lysis with 1% saponin to release intracellular bacteria and plated on TSB plates (Teknova, Hollister, CA). Bacterial colonies were counted next day after incubation overnight at 37 °C.

### Immunofluorescence

For staining of bacteria, HMEECs were cultured in 8-well chamber slides and infected with *S. aureus* for varying time periods. After incubation, cells were washed three times with PBS buffer and then fixed and permeabilized with BD cytofix and cytoperm reagent (BD Biosciences, San Jose, CA) for 30 min. After washing, the cells were blocked with 3% normal goat serum (NGS) (Sigma, St. Louis, MO) for 20 min and then incubated with FITC conjugated anti-*Staphylococcus aureus* antibody (1/100) (Abcam, Cambridge, MA) for 45 min. After washing, cells were mounted in an antifade Vectashield solution containing 4,6-diamidino-2-phenylindole (DAPI) (Vector Laboratories, Burlingame, CA). In some experiments, cells were stained for lipid rafts using anti-Flotillin 1 antibody (1/100) (Sigma, St. Louis, MO) followed by staining with Alexa Fluor 568 secondary antibody (1/500) (Life Technologies, Carlsbad, CA). The cells were viewed with a Zeiss LSM 710 microscope (Carl Zeiss, Germany) and images were assembled using Adobe photoshop 7.0.

### Statistical analysis

Statistical significance was determined by a paired, two-tailed Student’s t test or ANOVA using SPSS 15.0 software. Values of P < 0.05 were considered to be statistically significant.

## Supplementary information

Supplementary Material

## Data Availability

All data generated or analysed during this study are included in this article.
