# Supplementary material for: Otopathogenic Staphylococcus aureus Invades Human Middle Ear Epithelial Cells Primarily through Cholesterol Dependent Pathway
Source: Sci Rep. 2019 Jul 25;9:10777. doi: 10.1038/s41598-019-47079-7 (PMC6658548; doi:10.1038/s41598-019-47079-7)
Supplement: Supplementary file 1 — Supplementary Material [file 41598_2019_47079_MOESM1_ESM.pdf]

Supplementary information for

Otopathogenic *Staphylococcus aureus* Invades Human Middle Ear Epithelial Cells Primarily through Cholesterol Dependent Pathway

Rahul Mittal[1], Luca H. Debs[1], Amit P. Patel[1], Desiree Nguyen[1], Patricia Blackwelder[2,3], Denise Yan[1], Paulo H. Weckwerth[4], Xue Zhong Liu[1]

[1]*Department of Otolaryngology, University of Miami-Miller School of Medicine, Miami, FL, USA*

[2]*Center for Advanced Microscopy, Chemistry Department, University of Miami, Coral Gables, FL, USA*

[3]*RSMAS, University of Miami, Key Biscayne, FL, USA*

[4]*Health Sciences Department, University of Sagrado Coração, Bauru, SP, Brazil*

Address correspondence to: xliu@med.miami.edu and r.mittal11@med.miami.edu

Supplementary Figure 1

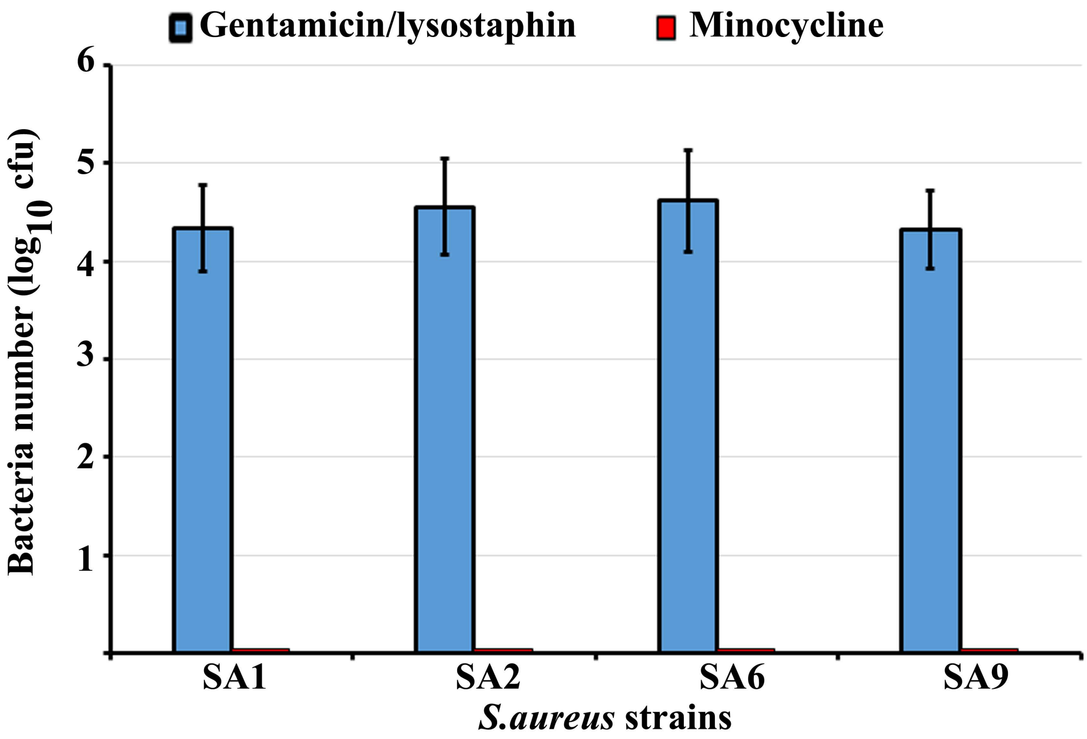

Supplementary Figure 2

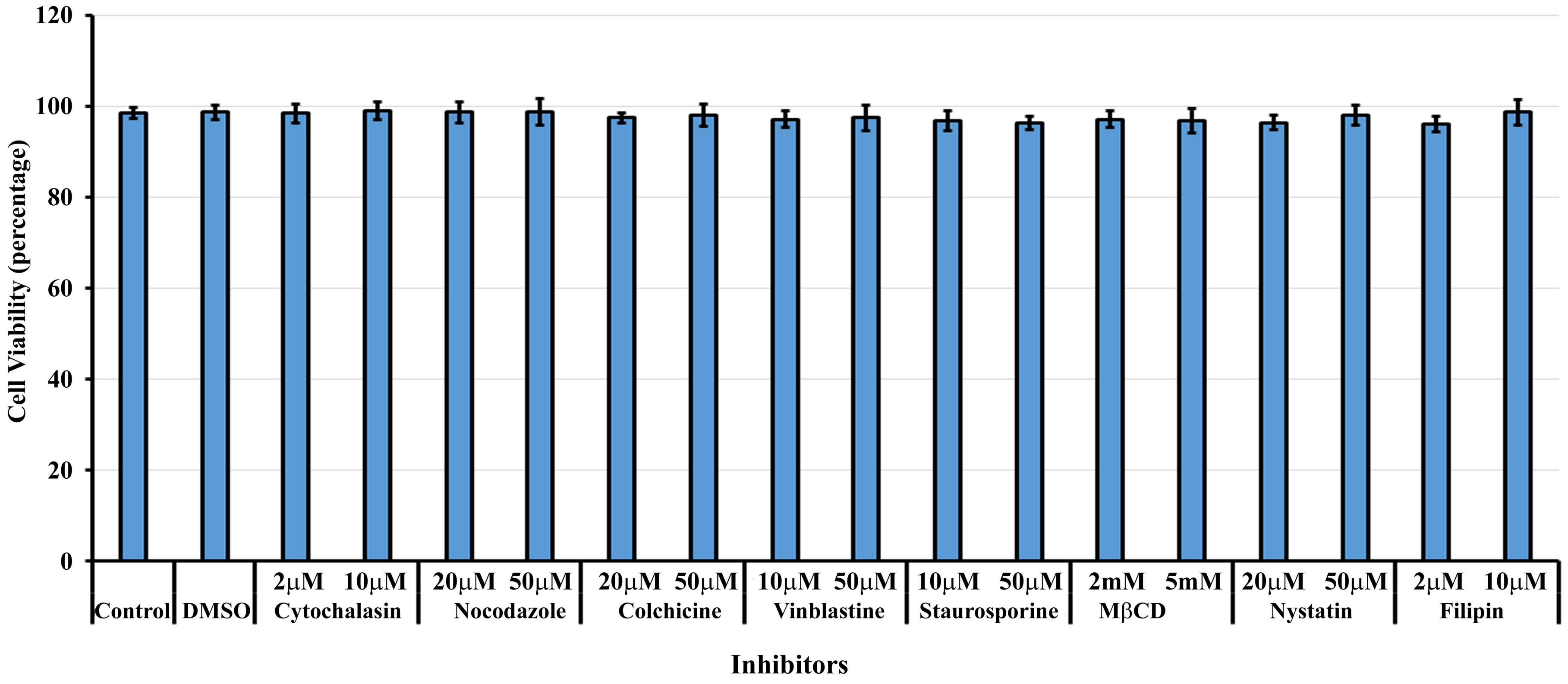

Supplementary Figure 3

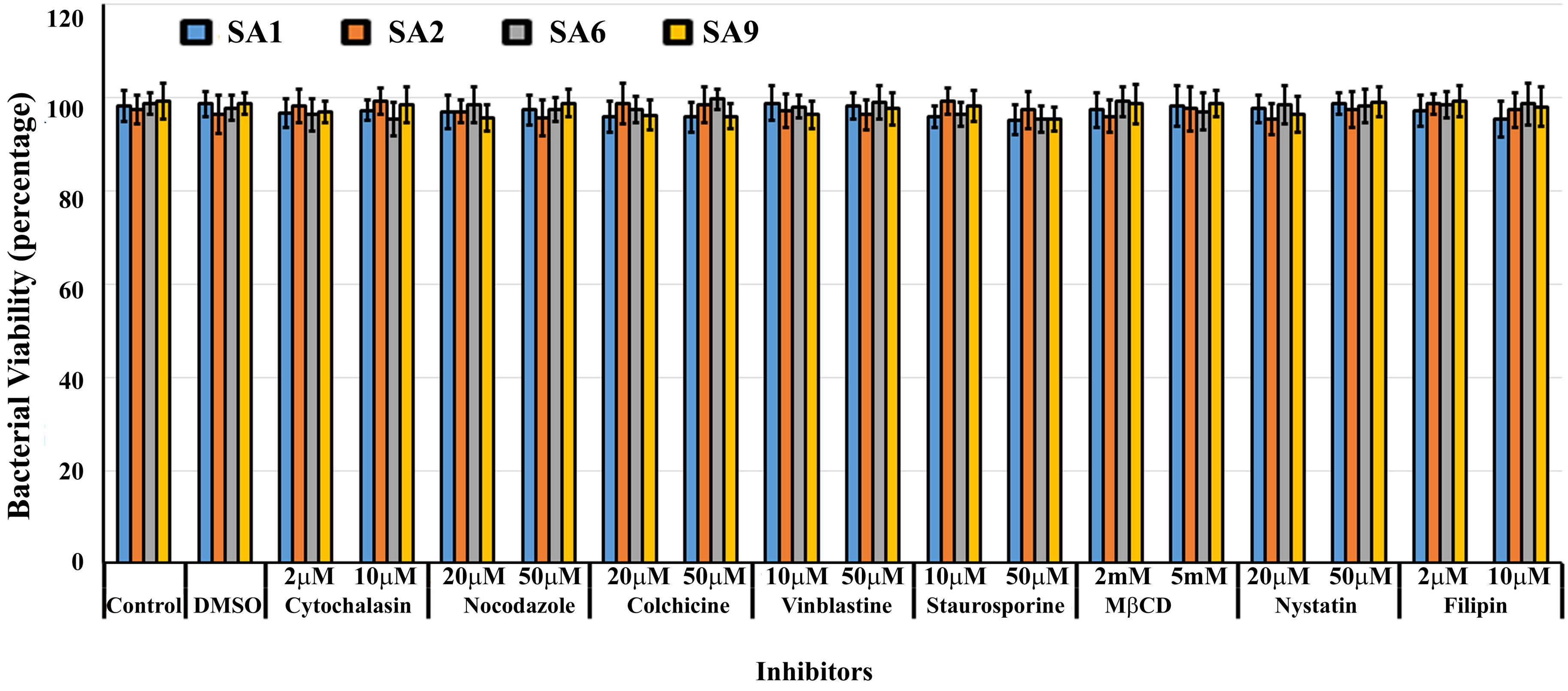

Supplementary Figure Legends

Supplementary Figure 1: Cell penetrating antibiotic kills intracellular bacteria. HMEECs were infected with *S. aureus* strains for 2h and then incubated with gentamicin and lysostaphin for 1h. After incubation, the cells were left either in medium or treated with cell penetrating minocycline antibiotic followed by washing and lysis with saponin to release intracellular bacteria. There were no viable bacteria recoverable from HMEECs treated with cell penetrating antibiotic minocycline. Data represents mean ± standard deviation and is representative of five different experiments carried in triplicate.

Supplementary Figure 2: Effect of inhibitors on cell viability. To determine the cytotoxicity of various inhibitors used in this study, HMEECs were incubated with different concentrations of inhibitors followed by washing and staining with Live/Dead cell viability kit. Results are expressed as percentage cell viability. Data represents mean ± standard deviation and is representative of four different experiments carried in triplicate.

Supplementary Figure 3: Effect of inhibitors on bacterial viability. The effect of inhibitors on *S. aureus* viability was assessed by incubating the bacteria with different concentrations of inhibitors followed by washing and staining with Live/Dead BacLight bacterial viability kit. Results are expressed as percentage bacterial viability. Data represents mean ± standard deviation and is representative of four different experiments carried in triplicate.
